# Dynamics of Early Establishment of SARS-CoV-2 VOC Omicron Lineages in Minas Gerais, Brazil

**DOI:** 10.3390/v15020585

**Published:** 2023-02-20

**Authors:** Mariane Talon de Menezes, Filipe Romero Rebello Moreira, Charles Whittaker, Franciele Martins Santos, Daniel Costa Queiroz, Victor Geddes, Paula Luize Camargos Fonseca, Jaqueline Góes de Jesus, Franciane Mendes-Oliveira, Valquíria Reis-Souza, Bibiana Santos, Danielle Alves Gomes Zauli, Aline Brito de Lima, Cristiane de Brito Mendonça, Luige Biciati Alvim, Joice do Prado Silva, Frederico Scott Varella Malta, Alessandro Clayton de Souza Ferreira, Nuno R. Faria, Ester Cerdeira Sabino, Renato Santana Aguiar

**Affiliations:** 1 Laboratory of Molecular Virology, Institute of Biology, Department of Genetics, Federal University of Rio de Janeiro, Rio de Janeiro 21941-901, Brazil; 2 MRC Centre for Global Infectious Disease Analysis, Imperial College London, London SW7 2BX, UK; 3 Laboratory of Integrative Biology, Department of Genetics, Ecology and Evolution, Institute of Biological Sciences, Federal University of Minas Gerais, Belo Horizonte 31270, Brazil; 4 Institute of Tropical Medicine, Faculty of Medicine, University of São Paulo, São Paulo 04023, Brazil; 5 Department of Infectious and Parasitic Diseases, Faculty of Medicine, University of São Paulo, São Paulo 04023, Brazil; 6 Mendelics Genomic Analysis, São Paulo 04023, Brazil; 7 Pardini Group, Research and Development Department, Belo Horizonte 31270, Brazil; 8 Department of Zoology, University of Oxford, Oxford OX3 7BN, UK; 9 D’OR Institute of Research and Teaching, Rio de Janeiro 21941-901, Brazil

**Keywords:** Omicron, SARS-CoV-2, genomic surveillance, variants of concern, viral evolution

## Abstract

Brazil is one of the nations most affected by Coronavirus disease 2019 (COVID-19). The introduction and establishment of new virus variants can be related to an increase in cases and fatalities. The emergence of Omicron, the most modified SARS-CoV-2 variant, caused alarm for the public health of Brazil. In this study, we examined the effects of the Omicron introduction in Minas Gerais (MG), the second-most populous state of Brazil. A total of 430 Severe Acute Respiratory Syndrome Coronavirus 2 (SARS-CoV-2) samples from November 2021 to June 2022 from Belo Horizonte (BH) city were sequenced. These newly sequenced genomes comprise 72% of all previously available SARS-CoV-2 genomes for the city. Evolutionary analysis of novel viral genomes reveals that a great diversity of Omicron sublineages have circulated in BH, a pattern in-keeping with observations across Brazil more generally. Bayesian phylogeographic reconstructions indicate that this diversity is a product of a large number of international and national importations. As observed previously, São Paulo state is shown as a significant hub for viral spread throughout the country, contributing to around 70% of all viral Omicron introductions detected in MG.

## 1. Introduction

The emergence and global spread of Severe Acute Respiratory Syndrome Coronavirus 2 (SARS-CoV-2) was the driver of one of the largest global health crises of the century, accounting for more than 6 million deaths worldwide (https://covid19.who.int/, accessed on 11 January 2023). Brazil was one of the countries impacted most by the Coronavirus disease 2019 (COVID-19), with disproportionately high numbers of cases and deaths (9.5% and 10.4% of the total, respectively, while the country harbors 2.7% of the global population) [1]. The first case of COVID-19 in the country occurred in late February 2020 in São Paulo state [2], later spreading to all other regions of the nation through multiple virus international introductions [3]. To date, SARS-CoV-2 has caused more than 36 million infections and 690,000 deaths in Brazil (https://covid.saude.gov.br, accessed on 11 January 2023).

Since the beginning of the COVID-19 pandemic, several SARS-CoV-2 variants have emerged and spread quickly throughout the world. Distinct phenotypes relating to the efficiency of diagnostic, immune escape potential, transmissibility, and disease severity has led to significant interest in monitoring and tracking particular lineages [4]. These are typically categorized by the World Health Organization (WHO) as variants of concern (VOC), variants of interest (VOI), and as variants under monitoring (VUM). The impact of the genetic changes on the virus properties and the risk that the variant represents to the world are used to establish these categories (https://www.who.int/activities/tracking-SARS-CoV-2-variants, accessed on 11 January 2023).

The global epidemic waves were shaped by the emergence of different VOCs: Alpha (Pango lineage B.1.1.7), first detected in UK in September 2020 [5]; Beta (Pango lineage B.1.351), first detect in South Africa in October 2020 [6]; Gamma (Pango lineage P.1) first detected in Brazil in November 2020 [7]; Delta (Pango lineage B.1.617.2), first detected in India in December 2020 [8]; and Omicron (Pango lineage B.1.1.529), first detected in South Africa in November 2021 [9]. More recently, the global pandemic landscape has been shaped by the emergence and spread of multiple Omicron sub variants, including BA.2, BA.3, BA.4, and BA.5, all characterized after the first Omicron (BA.1) description [6,9]. 

Omicron exhibits more than 60 mutations in relation to the original Wuhan-Hu-1 reference genome, with 32 of them in the receptor binding domain (RBD) of the spike protein, an essential domain for transmission and immune evasion properties [10]. The global spread of Omicron lineages has led to a resurgence of the pandemic in many countries, raising concerns among public health authorities [9,11]. In fact, numerous studies have demonstrated that Omicron has fitness advantages over the previously dominating Delta variant, such as infectivity, transmissibility, and escape from antibody recognition [10,12]. Even though a substantial percentage of the Brazilian population have received the full regimen of the SARS-CoV-2 vaccine (more than 80% with two vaccine doses, as available in https://conselho.saude.gov.br/vacinometro, accessed on 27 December 2022), the country has seen a significant rise in COVID-19 infections ever since VOC Omicron was first introduced in the country on November 30th of 2021 in São Paulo state.

Here, we conducted a population-based study in Belo Horizonte city (BH), the capital and the largest city in the Minas Gerais (MG) state, which accounts for about 10% of the state’s total population, in order to track the introduction and establishment of VOC Omicron. MG state is the second-most populous in Brazilian federal unit (over 21 million individuals) and shares borders with two of the largest Brazilian metropolises, Rio de Janeiro and São Paulo (https://www.ibge.gov.br, accessed on 27 December 2022) The state is also directly connected to three geographic Brazilian regions (Southeast, Northeast, and Centre-West), being an important hub for epidemiological surveillance activities. The genomic epidemiologic monitoring was carried out during two epidemic waves of SARS-CoV-2 in BH city, between epidemiological weeks (EWs) 44-2021 and 22-2022 (November 2021–June 2022). Through this effort, we sequenced 430 near-complete SARS-CoV-2 genomes. This study demonstrates the impact of Omicron introduction in a Brazilian context, with both genetic and epidemiological data supporting a chain of successive lineage replacement and virus introduction events involving Delta and Omicron (BA.1 and BA2) variants.

## 2. Materials and Methods

### 2.1. Study Population and SARS-CoV-2 Diagnostic

The study population is composed of RT-qPCR SARS-CoV-2 positive subjects tested at the Hermes Pardini Institute in Belo Horizonte between 1 November 2021 and 4 June 2022. Diagnosis was performed on nasopharyngeal specimens. Briefly, swabs were used to extract total viral RNA using an automated bead-based kit (KingFisher Flex System; ThermoFisher Scientific, Waltham, MA, USA), according to the manufacturer’s instructions. SARS-CoV-2 RNA was detected by RT-qPCR using iTaq Universal Probes One-Step Kit (Bio-Rad laboratories, Hercules, CA, USA) and SARS-CoV-2 (2019-nCoV) multiplex CDC RT-qPCR Probe Assay (Integrated DNA Technologies, Coralville, IA, USA), which targets the virus N1 and N2 genes. QuantStudio 5 (Thermo Fisher Scientific, Waltham, MA, USA) and CFX Opus 96 (Bio-Rad, Hercules, CA, USA) real-time devices were used for the RT-qPCR reactions. This study was approved by a Research Ethics Committee under protocol Certificado de Apresentação de Apreciação Ética (CAAE) 33202820.7.1001.5348.

### 2.2. Epidemiological Data

Epidemiological data, including COVID-19 positive cases and deaths for Belo Horizonte municipality were downloaded at https://sescloud.saude.mg.gov.br (acceded on 23 August 2022). Epidemiological analyses were executed in the R software (version 4.1.1) [13]. The EpiEstim package [14] was used to calculate the effective reproduction number, using a previously estimated serial interval for Omicron variants. The packages ggplot2 [15] and ggtree [16] were used to generate visualizations.

### 2.3. Multinomial Model

We used a previously published multinomial logistic regression model of competitive growth [17] to estimate the relative transmissibility of different variants of concern based on lineage frequency data derived from genomic sequencing of cases (described in further detail below). Here, we use the Delta variant-of-concern as our reference and estimate the relative transmissibility of BA.1 and BA.2, under the assumption that the generation time of these two lineages remained unchanged relative to Delta. The model was implemented in a Bayesian framework, with inference carried out using the probabilistic programming language Stan [18]. Parameter inference was based on 4 chains of 10,000 iterations, run in parallel, and with the initial 5000 iterations removed as burn-in.

### 2.4. Genome Sequencing

We selected 95 SARS-CoV-2 positive samples per month presenting the highest viral loads (Ct values below 30). A total of 571 samples were selected for sequencing using the Oxford Nanopore sequencing technology, corresponding to 0.5% to all Belo Horizonte SARS-CoV-2 positive cases in the study period. We employed a viral genetic material enrichment strategy with the Midnight RT-PCR Expansion kit (Nanopore Technologies, Oxônia, UK), as described previously [19].

First, cDNA synthesis on the isolated RNA was carried out using the LunaScript RT mastermix (New England Biolabs, Hitchin, UK). Next, the gene-specific multiplex PCR was carried out using the Midnight primer pools and Q5 high-fidelity polymerase (New England Biolabs, UK), producing 1200-bp amplicons that overlapped to cover the 30-kb SARS-CoV-2 genome. For library construction, the rapid barcoding kit (Nanopore Technologies, Oxônia, UK) was used, according to the manufacturer’s instructions. Finally, 800 ng of constructed library was loaded into R9.4.1 flow cells and sequenced on the Oxford Nanopore Minion Mk1C for 72 h.

### 2.5. Viral Genome Assembly and Classification

The Artic nCoV-19 workflow was used for consensus sequence generation (https://artic.network/ncov-2019/ncov2019-bioinformatics-sop, accessed on 25 August 2022). The pipeline performs a reference alignment of the basecalled reads against the specified reference sequence (NCBI id: NC_045512) using Minimap2 program [20] and executes variant calling with the Medaka software (https://github.com/nanoporetech/medaka, accessed on 25 August 2022). The median genome coverage of the consensus sequences generated was 94.9 percent. A total of 313 sequences had coverage > 95%. The obtained sequences with less than 75 percent coverage breadth were removed from downstream analysis. Consensus sequences were then classified using the Pangolin tool v.4.1.1 [21]. All generated genome sequences were made available at the Global Initiative on Sharing All Influenza Data (GISAID) databases (https://gisaid.org, accessed on 2 September 2022) (EPI_SET ID: EPI_SET_230113us; https://doi.org/10.55876/gis8.230113us accessed on 2 September 2022). Samples sequencing statistics and metadata can be found in Table S1 (Supplementary Materials).

### 2.6. Phylogenetic Analyses

#### 2.6.1. Maximum-Likelihood Phylogenetic Inference and Analysis of Temporal Signal

To characterize the evolutionary origins of Omicron sequences circulating in Belo Horizonte, we assembled separate datasets for variants BA.1 and BA.2. As background, these datasets included all BA.1 (NextStrain clade 21k) and BA.2 (NextStrain clade 21L) sequences from these variants NextStrain builds, respectively (captured on 18 August 2022, BA.1 = 4644, BA.2 = 4477). In addition, random samples of sequences from every Brazilian state were taken in proportion to the predicted weekly Omicron cases in each state during the study period, as per previous analysis [22]. Epidemiological data for this analysis were collected at https://opendatasus.saude.gov.br (accessed on 2 September 2022) database. This approach produced a BA.1 dataset of 9145 sequences, including 547 from MG (EPI_SET ID: EPI_SET_230112nt; https://doi.org/10.55876/gis8.230112nt accessed on 2 September 2022). For BA.2, 8225 sequences composed the final dataset, including 240 from MG (EPI_SET ID: EPI_SET_230112vd; https://doi.org/10.55876/gis8.230112vd accessed on 2 September 2022). The median genome coverage of the reference sequences included in datasets was 99.6 percent. Whenever sampling gaps were detected, all available data were included. Detailed calculations for the dataset composition are available in Table S2. For the recombinant sequences, we used a dataset with 800 sequences of different lineages (Delta, Omicron BA.1, Omicron BA.2, Gamma, XAG, XE, XF, XG, XL, XN, XQ, and XR recombinants) that has been previously published and used for XAG classification (GISAID Identifier: EPI_SET_220828ya; https://doi.org/10.55876/gis8.220828ya accessed on 2 September 2022) [23].

For each dataset, a maximum-likelihood (ML) phylogenetic reconstruction was performed using IQ-Tree program v2.1.2 [24] under the GTR+F+I+G4 nucleotide substitution model [25]. The Shimoidara–Hasegawa-like approximate likelihood ratio test (SH-aLRT) was used to measure phylogenetic uncertainty along tree branches [26]. The ‘---polytomy’ flag was used to collapse extremely short branches into unresolved nodes. Each tree was rooted manually by the oldest sequences present in the datasets. Then, trees were submitted to TempEst v1.5.3 [27] to identify samples with inconsistent temporal signals (outliers in the root-to-tip regression). Sequences were identified as outliers and excluded from the dataset if their residuals exceeded 1.5 times the interquartile range of the residuals distribution.

#### 2.6.2. Bayesian Molecular Clock Analyses

Given the sizes of the evaluated datasets, we performed time scale reconstructions with a fast Bayesian method that approximates the posterior distribution of dated trees with BEAST v.1.10.5 (pre-release 0.1). This method (BEAST Thorney), which has been previously used to infer large SARS-CoV-2 timescales [22,28], uses an alternative likelihood function based on a simple Poisson model that massively speeds up computations. The reconstructions were performed with a fixed evolutionary rate, 7.5 × 10^−4^ substitutions per site per year (s/s/y), consistent with previous studies [29,30]. Moreover, the selected rate is also coherent with the temporal scope of the datasets (approximately one year), considering previous observations on the time dependency of SARS-CoV-2 evolutionary rates [31].

To obtain independent assessments of timescales for lineages BA.1 and BA.2, standard Bayesian reconstructions were performed on subsets of both proportional datasets with BEAST v1.10.4 [32]. These subsets were composed of ten selected sequences per epidemiological week comprehended in the large datasets. Sequences presenting the smallest residuals in the root-to-tip regression within each week were selected, to assure that the temporal structure of the large dataset would be reflected in the subsets. These analyses used: I—the HKY+G4 substitution model [25,33], ii—the strict molecular clock model and iii—the skygrid tree prior [34] with monthly grids and a cut-off matching the x-axis intercept from the root-to-tip regression.

For each dataset, multiple Markov Chain Monte Carlo (MCMC) runs were performed and the software Tracer [27] was used to verify convergence and mixing of independent analysis (effective sample size > 200 for all parameters). Logs were combined with logcombiner [32]. This software was also used to sample 1000 trees of the combined posterior distribution of Thorney dated trees.

#### 2.6.3. Phylogeographic Inferences

To characterize the dynamics of the introduction of lineages BA.1 and BA.2 in MG, we performed phylogeographic reconstructions with discrete asymmetric models [35]. These models comprehended nine different states: four politically defined Brazilian regions (North, Northeast, Centre-West, South), four federal units in the Southeast Brazil border of MG state (Rio de Janeiro, São Paulo, Minas Gerais, and Espírito Santo), as well as international sequences. The selected discretization scheme considers that the numbers of cases and genomic surveillance capacity dramatically differ between federal units, with states in the Southeast frequently exhibiting a higher number of sequences than other regions in the country. In this sense, this scheme allows the estimation of the migration dynamics with a sensible number of states—facilitating computational feasibility—while allowing one to untangle the relative contribution of different regions, as well as RJ and SP, main hubs of SARS-CoV-2 transmission in Brazil [3,22], for the establishment of Omicron lineages in MG.

To map all transitions among locations, we used a robust counting method (Markov Jumps; [36]). For all datasets, independent analyses were run long enough to obtain good mixing and convergence. Afterwards, logs and trees were combined, as before. Finally, maximum clade credibility trees were inferred for both datasets with TreeAnnotator [32].

## 3. Results

### 3.1. SARS-CoV-2 Omicron Replacement of Delta in MG

**Figure 1:**
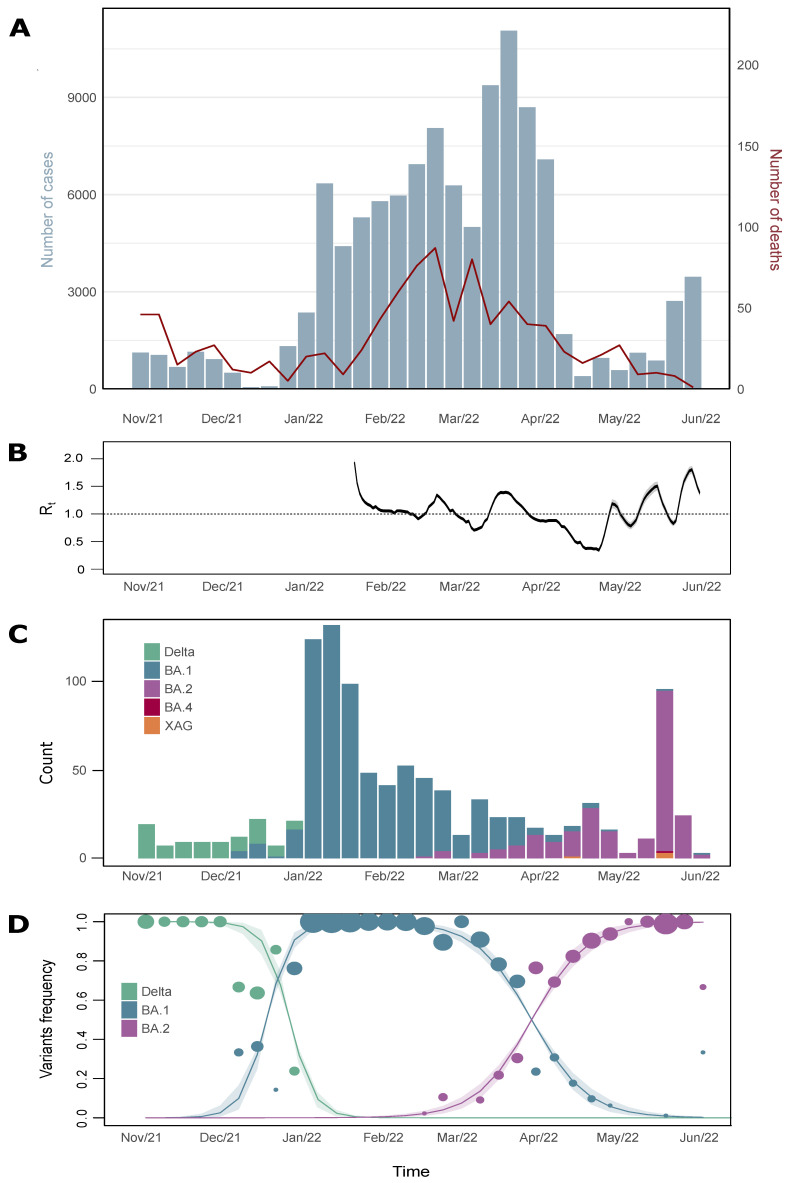
SARS-CoV-2 epidemiological monitoring during November 2021 and June 2022 (EWs 44-2021 and 22-2022); (**A**) Number of positive cases (blue bars) and deaths (red line) by COVID-19 reported in Minas Gerais state; (**B**) Reproductive number of virus infection during the Omicron transmission period in MG; (**C**) VOC prevalence profile in MG, according to sequences released in GISAID database; (**D**) Multinominal model analysis with the genome-released data during the study period in the state. The circles are proportional to genome numbers obtained for each variant. Line shadows are representative for 95% HPD.

SARS-CoV-2 infections in Brazil started to decline by the end of 2021. The same pattern was also seen in the state of Minas Gerais, where the lowest number of cases was registered in early December 2021 (EW 50–54 cases). The introduction and establishment of VOC Omicron in MG caused a massive rise in COVID-19 positive cases in early January 2022, reaching peak in late March 2022 (EW 12–11,057 cases), and a modest increase in mortality (Figure 1A). The reproductive number of the virus was above the critical threshold (Rt = 1) in several intermittent periods (maximum Rt = 1.8 on 29 May 2022), denoting intervals of sustained Omicron transmission in the state, consistent with the observed trends in the national incidence (Figure 1B).

To determine the prevalence and distribution of VOC Omicron in MG, we used the nanopore strategy to perform whole genome sequencing of 571 COVID-19-positive samples from residents of Belo Horizonte city, collected between 1 November 2021 and 4 June 2022. A total of 430 genome sequences with 75% > coverage breadth were generated and subsequently characterized in this effort (EPI_SET ID: EPI_SET_230113us; https://doi.org/10.55876/gis8.230113us accessed on 2 September 2022). Pangolin classification indicated the presence of variants Delta (*n =* 23), Omicron BA.1 (*n =* 229), Omicron BA.2 (*n =* 173), Omicron BA.4 (*n =* 1) and recombinant XAG (*n =* 4).

The 430 novel genomes here evaluated comprised 72% of all available SARS-CoV-2 genomes in GISAID of BH over the study period. The pangolin classification and lineages identification can be found in Table S1. One recombinant lineage, known as XAG, was identified in four samples in our study. The XAG recombinant is a product of BA.1 and BA.2 lineage recombination that has 6513-88392 nucleotide site as the likely recombination breakpoint. All four recombinant XAG sequences discovered in this work include unique nucleotide substitutions linked to the XAG lineage, such as the synonymous C2857T, A12334G, and C17502T changes and the nonsynonymous C5585A (L1774I) substitution. A maximum-likelihood phylogenetic analysis with a globally representative XAG dataset shows the new sequences cluster with previously described Brazilian sequences, composing a clade closely related to a sequence from the United States (Figure S1).

In early November 2021 (EW 44), the Delta variant comprised 100% of the cases. Omicron BA.1 variant was first detected in MG state in early December 2021 (EW 49). It took four weeks to comprise 100% of the sequences detected. The Omicron BA.2 variant was identified in our dataset for the first time in February 2022 (EW 07), and by the end of the study, the frequency of the variant was 66.6% (Figure 1C).

Statistical analysis performed with a multinomial model suggests the BA.1 variant was 2.59× more transmissible than the Delta variant. In the same way, Omicron BA.2 was estimated to be 1.5× more transmissible than BA.1. In this manner, the BA.2 variant was estimated to be approximately 3.9× more transmissible than the Delta variant (Figure 1D).

### 3.2. Phylogeographic Origins of VOC Omicron Lineages in Minas Gerais

**Figure 2:**
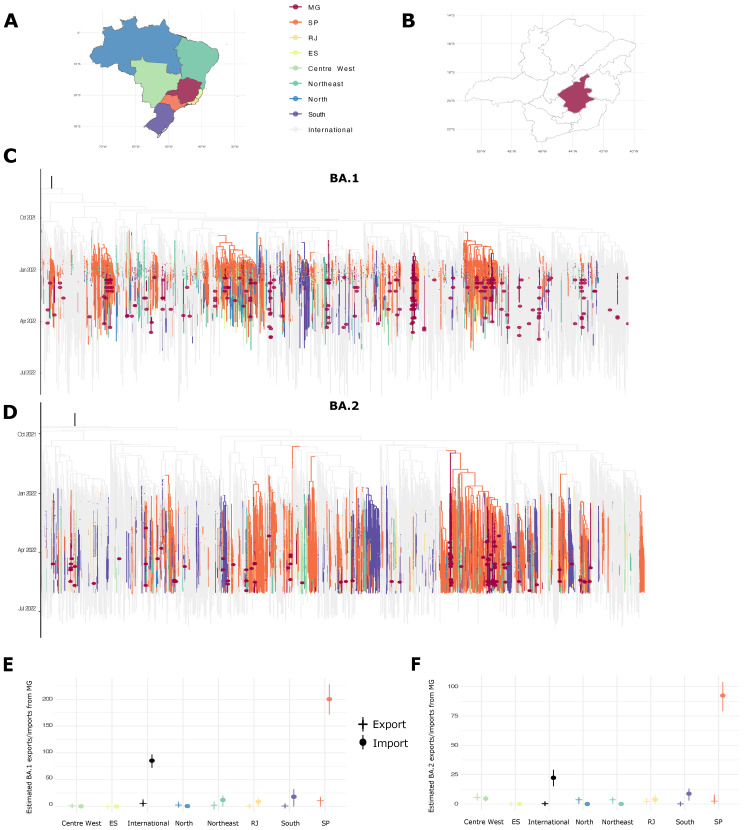
Omicron BA.1 and BA.2 phylogenetic reconstruction in Minas Gerais; (**A**) Map of Brazil colored by regions selected for phylogeography analysis: Minas Gerais state (MG), São Paulo state (SP), Rio de Janeiro state (RJ), Espírito Santo state (ES), and Centre-West, Northeast, North, and South Brazilian regions; (**B**) Map of MG state macro-regions, showing in dark red the metropolitan Belo Horizonte region; (**C**,**D**) Bayesian reconstruction demonstrates MG sequences distributed along the tree, suggesting different imports in the region for BA.1 and BA.2 lineages. Dark red circles represent sequences generated in this study. Tree branch colors are corresponding to geographical sequence locations as shown in the map; (**E**,**F**) Regional estimates of the imports and exports of the BA.1 and BA.2 viruses to/from MG, regionally colored as the map.

Maximum-likelihood phylogenetic analyses were performed with Omicron BA.1 and BA.2 datasets separately. For both variants, the novel sequences clustered along the tree in different branches, suggesting different introductions in the state and high viral diversity (Figure 2C,D). Strong temporal signals were observed in both trees (R = 0.33 and 0.17, Slope = 6.3 × 10^−4^ and 4.2 × 10^−4^, respectively), appropriate for a further Bayesian analysis.

Bayesian molecular-clock-based analyses estimated BA.1 to have emerged in the world in late August 2021 (95% HPD: 28 July 2021–21 September 2021) and by early October the virus was already circulating in Brazil. Our analysis demonstrates that around 7.9% of the worldwide BA.1 introductions to Brazil (*n =* 1064; 95% HPD: 1012–1111) were made to the state of Minas Gerais (*n =* 85; 95% HPD: 72–97). In parallel, we identified 156 export events from Brazil to other countries in our dataset (95% HPD: 130–184), but MG low frequency international exports were significant uncertainty.

Brazilian interstate transitions were estimated using different Brazilian regions (North, Northeast, Centre-West, and South) and Southeast (where MG is located) states stratified (Rio de Janeiro, São Paulo, and Espírito Santo) (Figure 2A). The region which contributed the most BA.1 imports to MG was São Paulo state, where the BA.1 was first identified (*n =* 200; 95% HPD: 172–228) (Figure 2C,E). The remaining locations contributed to a much smaller number of introductions, often including zero in the HPD for the number of transitions (results summarized in Table S3). Export events from MG were also detected, though with considerable uncertainty (Figure 2E, Table S3).

The time estimated for the BA.2 ancestor was early September 2021 (95% HPD: 28 August 2021–6 October 2021) and, as Omicron BA.1, the variant was already circulating in Brazil in early October (Figure 1D). When compared with Omicron BA.1 variant, BA.2 variant shows fewer international viral introductions in Brazil (*n =* 526; 95% HPD: 500–550), 4.1% of these were to MG (*n =* 22; 95% HPD: 15–29) (Figure 1F). BA.2 also shows fewer export events (*n =* 80; 95% HPD: 63–95) in our model.

São Paulo state was also the major contributor to MG BA.2 introductions, accounting for 70.7% (*n =* 92; 95% HPD: 79–104) of all introductions, followed by South (*n =* 8; 95% HPD: 3–13), Centre-West (*n =* 4; 95% HPD: 2–7), and Rio de Janeiro (*n =* 4; 95% HPD: 0–8). Other importation and exportation routes have shown limited statistical support (Table S3). Exports to the Centre-West were an exception to this pattern (*n =* 5.5, 95% HPD: 3–9). Together, those results reinforce the international introduction of Omicron VOC in MG state followed by interstate transmission showing the importance of domestic control in the transmission and dispersion of COVID-19 in Brazil.

## 4. Discussion

The state of MG experienced a rapid replacement of the Delta by Omicron variants in early 2022, which was accompanied by an increase in the incidence of COVID-19. Omicron’s introduction, however, caused only a modest rise in the number of deaths, most likely due to the high vaccination status reported for the state (88.22% of population with the first vaccine dose, 83.40% with the second dose, 63.45% with the third dose and 41.56% with the fourth dose; at https://coronavirus.saude.mg.gov.br/vacinometro, accessed on 11 November 2022). The same trend was observed in the entire Brazilian territory and worldwide (https://covid19.who.int/, accessed on11 November 2023). In contrast to initial concerns of Omicron immunological vaccine escape [37,38], vaccination has been linked to lower death and hospitalization rates in communities with high vaccine coverage, such as Brazil [39].

Joint analysis of incidence and genetic data suggests the emergence and transmission of Omicron variants in MG resulted in an increase in transmissibility, as measured by the effective reproductive number (Rt). Throughout the study period, Rt oscillated between supercritical (>1) and subcritical (<1) values (ranging between 0.1–1.8 over the study period) and presented intervals above the critical threshold, indicating moments of sustained virus transmission, driving epidemic growth in early 2022. One limitation of our analysis is related to the lack of notifications in late December 2021 due to the end of year holidays, which possibly impacted the Rt estimate for early January 2022.

We used a multinomial logistic model to obtain transmissibility advantage estimates, as performed previously [17]. We estimate BA.1 to be 2.59 times more transmissible than VOC Delta, while BA.2 was only 1.5 times more transmissible than BA.1. These results are consistent with the relative speed of lineage replacement events observed in lineage frequency data, with BA.1 achieving 100% prevalence in 5 weeks, while for BA.2, replacement took 12 weeks after first detections. Indeed, our findings are consistent with a number of studies which similarly highlight a transmissibility advantage for the VOC Omicron over Delta [12,40,41].

Brazil represents an important site in the global dissemination of variants—indeed, the Omicron variant was initially introduced to South America through Brazil, from where the variant was conveyed to other countries on the continent [30]. Despite the high number of COVID-19 cases and the country’s substantial contribution to the outbreak, Brazil has significantly less SARS-CoV-2 genomic monitoring than other South American countries (three sequences per 10,000 cases) [31]. Our genomic surveillance was able to detect the XAG recombinant and Omicron BA.4 lineages in our population survey, with a low frequency. Omicron BA.4 was first detected in South Africa [42] and rapidly spread around the world. Here, we identify one sequence of Omicron BA.4 in EW 20-2022 (May 2022). XAG recombinant virus is a variant product of genome recombination of BA.1 and BA.2 variants co-infection first detected in Brazil in May 2022 [23,43]. In the present study, the XAG recombinant variant was first seen in early April, after the co-circulation of variants BA.1 and BA.2 in Minas Gerais. More studies are necessary to evaluate the impact of the introduction of these new variant in shaping the evolution of the COVID-19 epidemic in Brazil. Altogether, our findings underscore how crucial genomic surveillance is to Brazil’s ability to track emerging viral lineages.

With almost 21 million residents, the state of Minas Gerais is the second most populous in Brazil. The state occupies an important geographical aspect since it shares borders with several Brazilian regions, including the Southeast, Northeast, and Centre-West. São Paulo and Rio de Janeiro, two of Brazil’s largest states, are bordered by MG. Both of these states have been shown to be essential for epidemic expansion and dispersion of COVID-19 [3,22,44,45,46]. This study demonstrates an important role in São Paulo state in virus dissemination for MG (almost 70% of all state introductions), related to several phylogenetic clades. In contrast, Rio de Janeiro was the major contributor for VOC Delta MG introductions, accounting for three-quarters of all state introductions [22]. We were unable to find any patterns in either terrestrial or aerial human movement that might explain this change in viral spread. However, Rio de Janeiro state registered a peak of SARS-CoV-2 positive cases in November 2021 (the moment when VOC Delta was prevalent in the Brazilian epidemic). On the other hand, São Paulo state experienced the highest numbers of positive cases in January 2022, after the VOC Omicron introduction (https://covid.saude.gov.br, accessed on 20 January 2023). These two states experienced COVID-19 outbreaks in distinct pandemic waves, which most likely caused a founder effect and affected the viral spread pattern in Brazil. Indeed, VOC Delta was introduced into MG less frequently than Omicron, indicating greater viral diversity of Omicron variant in the state. Our findings support the necessity of tracking interstate human mobility, particularly between the Southeastern states.

International introductions were the second-most important driver for Omicron establishment in MG. This was even more evident for the Omicron BA.1 scenario than for the BA.2 variant. Increased international travel and a relaxation of non-pharmaceutical interventions (NPI) against COVID-19, such as the usage of masks, may be contributing factors to the rise in world-wide viral diversity. International introductions were even more evident for BA.1 than BA.2; this profile could be related to holidays during which BA.1 variant were introduced in Brazil. Studies have previously linked this time of year with an increase in human mobility and COVID-19 positive cases [4,47]. Traveler-based genomic surveillance at airports showed to be an important tool for early lineage detections and control [48], our study emphasizes the importance of this control in Brazilian airports.

## 5. Conclusions

Overall, this study reports results from a SARS-CoV-2 genomic surveillance initiative in a crucial metropolis in Brazilian territory (Belo Horizonte city, MG state). Integrating genomic and epidemiological data for the state, we observed rapid variant turnovers associated with increases in incidence. The subsequent introductions of distinct Omicron lineages (BA.1 and BA.2) were responsible for the maintenance of a high number of COVID-19 cases in the state during the study period. We were also able to identify few sequences of BA.4 and XAG lineages, however, continuous surveillance is necessary to assess the public health impact of these new variants in MG. Furthermore, a strong epidemiological connection between MG and SP was revealed by our phylogeographic reconstructions. In the same way, international movement was also responsible for the great viral diversity found. Despite the effort to generate a proportionate dataset that accurately represented SARS-CoV-2 diversity in Brazil, this analysis has limitations, since the number of positive cases sequenced are still low and the computational feasibility to handle much larger datasets is still an issue. Although the exact number of imports and exports is certainly an underestimate, our analysis reveals an underlying transmission patterns, important for public health agencies to detect the main sources of viral lineages in the state. Finally, we demonstrate that despite the rapid epidemic growth caused by VOC Omicron, it did not result in an increase in fatalities and hospitalizations in MG (https://coronavirus.saude.mg.gov.br/dadosabertos, accessed on 27 December 2022). This is a result of the development of the vaccination program in the country, supporting the relevance of vaccines as key instruments to protect global health.

## Data Availability

All consensus genome sequences characterized in this study have been deposited on GISAID and are publicly available (EPI_SET ID: EPI_SET_230113us; https://doi.org/10.55876/gis8.230113us accessed on 2 September 2022).

## Supplementary Materials

The following supporting information can be downloaded at: https://www.mdpi.com/article/10.3390/v15020585/s1, Table S1: New genomes’ sequences metadata and pangolin classification; Table S2: Dataset composition for Phylogeographic inferences; Table S3: Number of viral transitions calculated in phylogeographic reconstruction; Figure S1: XAG recombinant sequences phylogenetic reconstruction. Maximum-likelihood phylogenetic reconstruction using 800 SARS-CoV-2 genomes representative of different viral lineages and recombinants. Blue square represents XAG recombinant clade (a). Zoom of phylogenetic clade where the four XAG sequences generated in this study (red circles) clustered with previously described Brazilian sequences, colored by region (b).
