# Supplementary material for: Dynamics of Early Establishment of SARS-CoV-2 VOC Omicron Lineages in Minas Gerais, Brazil
Source: Viruses. 2023 Feb 20;15(2):585. doi: 10.3390/v15020585 (PMC9962645; doi:10.3390/v15020585)

XAG recombinant sequences phylogenetic reconstruction

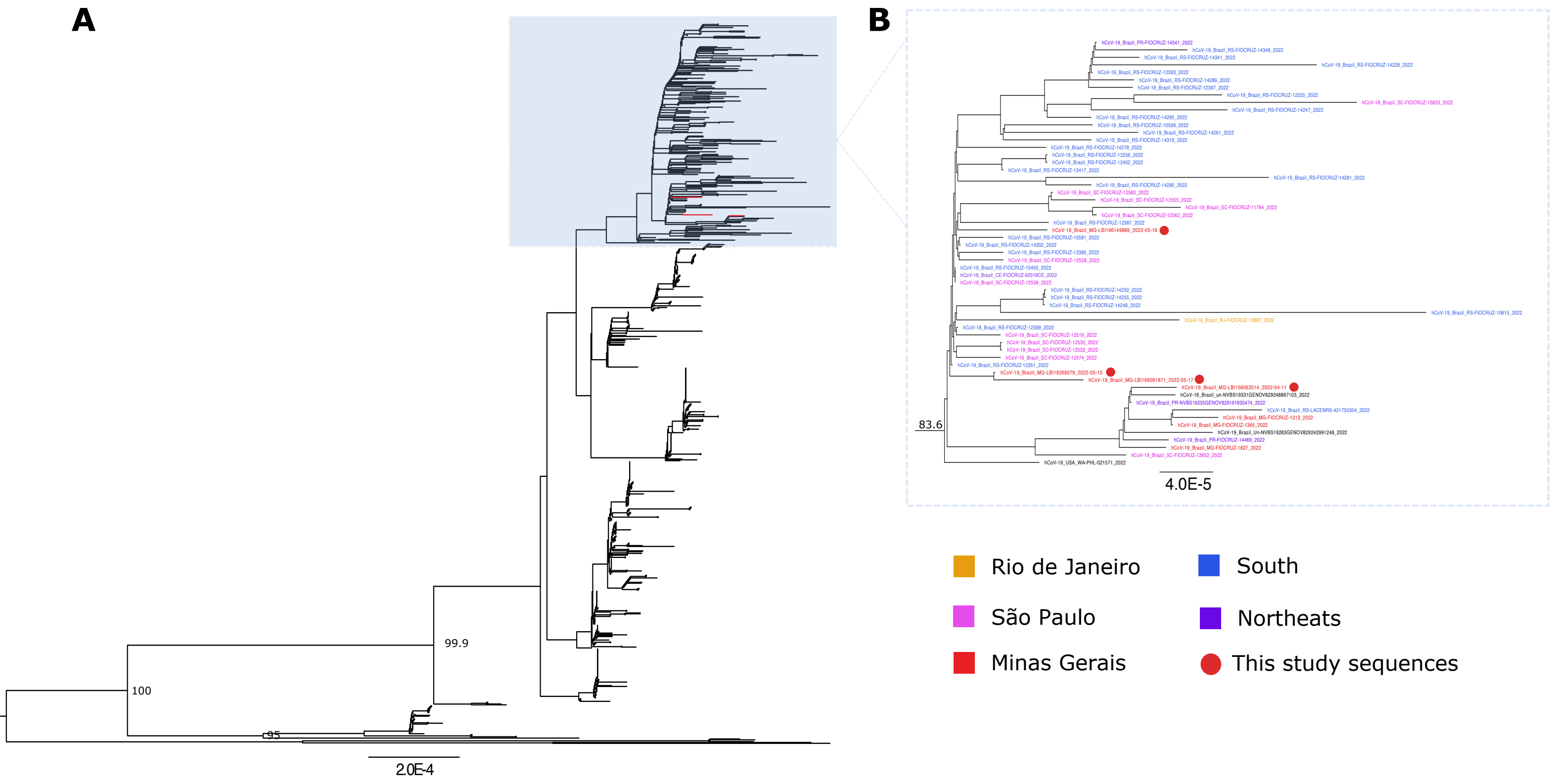

Supplement: Supplementary file 1 [file viruses-15-00585-s001.zip › Fig_Sup_XAG_review.pdf]
